# Clinical practice guideline for the management of lipids in adults with diabetic kidney disease: abbreviated summary of the Joint Association of British Clinical Diabetologists and UK Kidney Association (ABCD-UKKA) Guideline 2024

**DOI:** 10.1186/s12882-024-03664-1

**Published:** 2024-07-06

**Authors:** Sagen Zac-Varghese, Patrick Mark, Steve Bain, Debasish Banerjee, Tahseen A. Chowdhury, Indranil Dasgupta, Parijat De, Damian Fogarty, Andrew Frankel, Gabrielle Goldet, Janaka Karalliedde, Ritwika Mallik, Rosa Montero, Adnan Sharif, Mona Wahba, Ketan Dhatariya, Kieran McCafferty, Eirini Lioudaki, Peter Winocour

**Affiliations:** 1 East and North Herts NHS Trust, Hertfordshire, UK; 2 University of Glasgow, Glasgow, UK; 3 Swansea University, Swansea, UK; 4 George’s Hospital, London, UK; 5 Royal London Hospital, London, UK; 6 Heartlands Hospital, Birmingham, UK; 7 Birmingham City Hospital, Birmingham, UK; 8 Belfast Health and Social Care Trust, Belfast, Northern Ireland; 9 Imperial College London, London, UK; 10 Guy’s and St Thomas NHS Foundation Trust, London, UK; 11 Barts Health NHS Trust, London, UK; 12 University of Birmingham, Birmingham, UK; 13 Helier Hospital, Carshalton, UK; 14 Norwich University Hospital, Norfolkand, UK; 15 Queen Mary University of London, London, UK; 16 University College Hospital, London, UK; 17 Kings College London, London, UK

**Keywords:** Diabetic kidney disease, Lipid management, Diabetes, Lipids, Chronic kidney disease, Nephropathy

## Abstract

The contribution of chronic kidney disease (CKD) towards the risk of developing cardiovascular disease (CVD) is magnified with co-existing type 1 or type 2 diabetes. Lipids are a modifiable risk factor and good lipid management offers improved outcomes for people with diabetic kidney disease (DKD).

The primary purpose of this guideline, written by the Association of British Clinical Diabetologists (ABCD) and UK Kidney Association (UKKA) working group, is to provide practical recommendations on lipid management for members of the multidisciplinary team involved in the care of adults with DKD.

## Introduction

People with diabetes and chronic kidney disease (CKD) are at increased risk of developing cardiovascular disease (CVD). Lipids are a modifiable risk factor and good lipid management offers improved outcomes for people with diabetic kidney disease (DKD).

Lipid management should be considered alongside lifestylemeasures and management of blood pressure, weight, glycaemia, smoking cessation, and thrombotic risk. Attention should also be paid to the newer pillars of care for those with DKD, including sodium glucose co-transporter 2 inhibitors and non-steroidal selective mineralocorticoid receptor antagonists. These other aspects of DKD management are addressed in the Joint Association of British Clinical Diabetologists (ABCD) and UK Kidney Association (UKKA) guidelines [1, 2].

The primary purpose of this guideline is to provide practical recommendations on lipid management for members of the multidisciplinary team involved in the care of adults with DKD. This guideline covers: what to measure, frequency of monitoring, who to treat, treatment targets, what to use and, when to stop treatment.

**Fig. 1:**
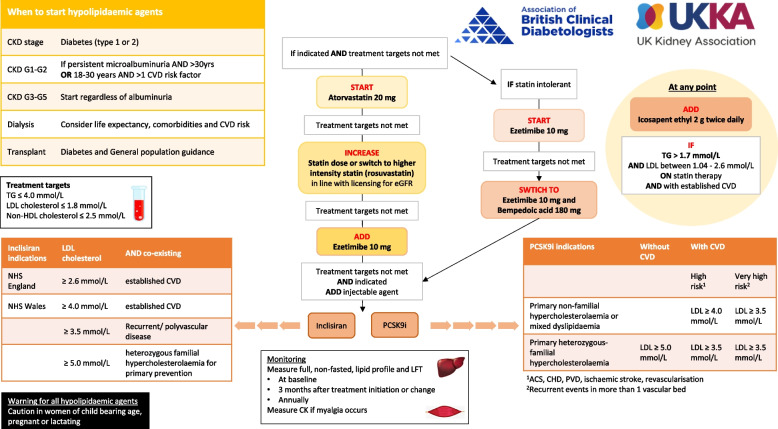
^Summary recommendations of the ABCD UKKA guideline for the management of lipids in diabetic kidney disease^

Figure 1 is a summary of the treatment pathway and recommendations (Summary recommendations of the ABCD UKKA guideline for the management of lipids in diabetic kidney disease).

The full guideline with explicit rational for recommendations can be found at https://abcd.care/position-papers.

### Methodology

This guideline is based on the opinion of the ABCD and UKKA working group and is an update of previous 2021 and 2017 guidance. This 2024 update is based on searches conducted between January 2020 and March 2023. We searched PubMed, the Cochrane database of systematic reviews and hand searched reference lists and articles identified by ABCD-UKKA writing group members.

Search terms used were ‘diabetes’, ‘lipids’ AND ‘chronic kidney disease/ nephropathy’. We also reviewed all related guidelines from the National Institute for Health and Care Excellence (NICE), UKKA, Kidney Disease Improving Global Outcomes (KDIGO), the European Renal Association Best Practice Guidelines, and the American and European Diabetes Associations.

The recommendation grades range from 1 (strong recommendation) to 2 (weak recommendation), and the corresponding evidence quality is: A (high-quality evidence), B (moderate-quality evidence), C (low-quality evidence), and D (very low-quality evidence).

Standard lipid abbreviations are used: total cholesterol (TC), chylomicron (CM), high density lipoprotein (HDL), low density lipoprotein (LDL), intermediate density lipoprotein (IDL), lipoprotein (a) (Lp(a)) and triglycerides (TG).

### Recommendations for lipid measurement and frequency of monitoring

There is clear evidence relating LDL cholesterol levels to atherosclerotic CVD (ASCVD) risk and evidence with regard to reducing LDL cholesterol levels and reducing ASCVD risk [3, 4]. There is also a relative risk attributable to non-HDL cholesterol (calculated as TC minus HDL cholesterol). A meta-analysis of people treated with statins suggested that non–HDL cholesterol may be a better predictor of coronary artery disease (CAD) risk than LDL cholesterol, possibly reflecting the additional impact of larger, atherogenic, TG rich molecules and the loss of benefit of higher HDL cholesterol levels [5].

In the past, fasting lipid profiles were recommended. However, obtaining fasting samples can be problematic and inconvenient, leading to delays in medication and disruption of glycaemic control. Large population studies show that there are only minor differences between fasting and non-fasting LDL and TG levels. In addition, non-fasting lipid levels correlate well with cardiovascular outcomes. National UK lipid guidelines (NICE) and European Society of Cardiology and European Atherosclerosis Society (ESC/EAS) guidelines also advocate the measurement of a non-fasting lipid profile.

Kidney transplant recipients have a high prevalence of dyslipidaemia. Immunosuppressive therapy, specifically corticosteroids, ciclosporin, sirolimus and everolimus, contributes to this [6]. Lipid assessment should be performed once immunosuppressive drug dosing is stable and the risk of acute rejection requiring corticosteroids has fallen. This is likely to be achieved 3 months post transplantation at the earliest.

People on dialysis represent a diverse group. Within this group, there are people who are continued on lipid-lowering agents and others where this is inappropriate. In addition, some people on dialysis will progress to receive a kidney transplant. Where it is of benefit, i.e. where it would change management, annual screening can be continued within this group.

There is marked variation between current guidelines regarding monitoring lipid profiles in DKD. We feel that annual screening is a reasonable approach. It is also acceptable to monitor more frequently if this influences management.

We recommend that a non-fasting full lipid profile (TC, non-HDL, HDL, LDL cholesterol and TG) is performed at least annually in stage G1-5 DKD, including post kidney transplantation and, where appropriate, in dialysis, stage G5d.

LDL cholesterol is calculated (using the Friedewald formula) and requires a fasting sample if TG levels are > 4.5 mmol/L. In hypertriglyceridaemia (> 4.5 mmol/L), we recommend measuring fasting profiles (Grade 1B).

We suggest review of the lipid profile on commencement or change of modality of renal replacement therapy (dialysis or kidney transplantation) (Grade 2D).

Following kidney transplantation, we suggest that lipid status be assessed once the immediate post-operative period has passed (typically 3 months post transplantation) (Grade 2D).

### Recommendations for lipid management in type 1 diabetes

Outcome studies of lipid lowering in people with type 1 diabetes and DKD are lacking. However, younger people with type 1 diabetes and persistent albuminuria have a substantially elevated lifetime CVD risk and this would be the basis for statin initiation. The principle of identifying exaggerated lifetime risk beyond the initial decade of treatment was clearly outlined in the Joint British Societies (JBS) 3 guidelines [7]. While the absolute risk for young people (aged 18 to 30 years) with DKD may be low, there is a high relative risk. There is no evidence base to support initiation of statins in type 1 diabetes aged < 18 years, or in newly diagnosed type 1 diabetes aged ≤ 30 years without any additional risk factors.

Where trials of people with type 1 diabetes and DKD are lacking, it is reasonable to extrapolate general population data and use CKD stage (Table 1) as a CVD risk equivalent. In CKD stages G3–5, the elevated risk of CVD justifies the initiation of lipid-lowering agents, notwithstanding the additional impact of type 1 diabetes in elevating this risk.

**Table 1:** Nomenture and categories of estimated glomerular filtration rates (eGFRs) and albuminuria. Adapted from KDIGO [8]

**GFR category**	**eGFR (ml/min/1.73m**^**2**^**)**
G1	≥ 90
G2	60–89
G3a	45–59
G3b	30–44
G4	15–29
G5	< 15
**Albuminuria category**	**Albumin to creatinine ratio (ACR) mg/mmol**
A1	< 3
A2	3–30
A3	> 30

Where the question arises whether or not to start those on dialysis, CKD stage G5d, on lipid-lowering agents, consideration should be placed on the likelihood of transplantation, life expectancy and the risk benefit ratio. In most cases, we do not recommend initiating lipid-lowering therapy in those on dialysis.

We suggest that in type 1 diabetes and stage G1–2 DKD, lipid-lowering therapy is commenced in the following categories:

- People aged > 30 years with persistent albuminuria, category A2-3 (Grade 2D).
- People aged between 18 to 30 years with persistent albuminuria, category A2-3, and ≥ 1 additional CVD risk factor (Grade 2D).

We recommend that in type 1 diabetes and stage G3–5 DKD, regardless of albuminuric status, lipid-lowering therapy is commenced (Grade 1C).

In people with type 1 diabetes on dialysis, stage G5d DKD, we do not recommend initiation of lipid lowering therapy. However, we suggest that in certain cases, e.g. in those where transplantation is a likely outcome and where dialysis is temporary, initiation might be beneficial and may be considered (Grade 2D).

### Recommendations for lipid management in type 2 diabetes

Until relatively recently, type 2 diabetes has been considered a CVD risk equivalent. It is now clear that diabetes per se is not a CVD risk equivalent [9]. Rather, certain characteristics are required to escalate CVD risk, most notably longer duration of diabetes and/ or the presence of albuminuria [10–12]. In addition, CKD, based on reduced GFR, also enhances CVD risk [9, 12]. Thus, the combination of type 2 diabetes with albuminuria, stage G3 CKD or higher substantially increases the risk of CVD [9].

There have been several large-scale prospective CVD outcome studies involving people with type 2 diabetes and CKD. The Cholesterol Treatment Trialists’ (CTT) Collaboration database, established in 1994, includes individual participant data from statin trials with at least 1,000 participants with ≥ 2 years of follow up. In the 2008 CTT meta-analysis of outcomes in over 18,000 people with diabetes from 14 randomised trials of statin therapy, a 1 mmol/L reduction in LDL cholesterol reduced the combined endpoint of CHD death and non-fatal MI by 22%, CVD events by 21%, vascular death by 13% and all-cause death by 9%. Coronary revascularisation was reduced by 25% and stroke by 21% [4].

A further CTT meta-analysis (2016) of data from 28 trials (*n*= 183,419; 35,781 with diabetes), confirmed that statins reduce the risk of a first major vascular event by 21% per mmol/L reduction in LDL cholesterol [13]. The CTT looked at risk ratios in sub-divisions of participants stratified by eGFR (≥ 60, 45– < 60, 30– < 45, < 30 and dialysis). Smaller effects were seen as eGFR declined with little evidence of benefit seen in dialysis [13].

These meta-analyses demonstrate the efficacy of statins as primary prevention. There is limited data in young people with type 2 diabetes. However, considering the elevated lifetime risk within this cohort, we have suggested that this younger cohort receives treatment.

We recommend that in people with type 2 diabetes with stage G1–2 DKD, lipid-lowering therapy is commenced in the following categories:

- People aged > 30 years with persistent albuminuria, category A2-3 (Grade 1C).
- People aged between 18 to 30 years with persistent albuminuria, category A2-3, and ≥ 1 additional CVD risk factor (Grade 1D).

We recommend that lipid-lowering therapy with statins should be considered in people with stage G3–5 DKD regardless of albuminuric status (Grade 1B).

In people with type 2 diabetes on dialysis, stage G5d DKD, we do not recommend initiation of lipid lowering therapy. However, we suggest that in certain cases, e.g. in those where transplantation is a likely outcome, initiation might be beneficial and may be considered (Grade 2D).

### Recommendations for lipid management in ESKD and dialysis

People with ESKD are at dramatically increased risk of premature CVD, 5–20 times that of the general population. However, the relationship between cholesterol and CVD risk is not clear and there is a ‘J’ shaped relationship between cholesterol and mortality, possibly driven by malnutrition or inflammation being associated with lower serum cholesterol levels.

Commencement of renal replacement therapy (dialysis or transplantation) for ESKD is associated with the need for major lifestyle changes including dietary and fluid restrictions, hospital attendance and medication. For some, continuation of lipid-lowering therapy may be inappropriate.

As discussed earlier, the 2016 CTT meta-analysis confirmed that overall, statins reduce the risk of a first major vascular event; however, smaller effects were seen as eGFR declined with little evidence of benefit seen in dialysis [13]. It is not clear if the reduced efficacy of statins in ESKD is due to the reduced proportion of people with atherosclerotic coronary heart disease or, due to a misclassification of deaths partly based on the difficult of interpreting raised troponins in this group [13].

Although clear evidence of benefit has not been demonstrated in trials of lipid-lowering therapy in people with diabetes on dialysis, currently, there are no convincing data to suggest harm in using lipid-lowering therapy. Many physicians continue lipid-lowering therapy in people on dialysis. However, it is uncommon for lipid-lowering therapy to be initiated in this group.

### Recommendations for lipid management post-transplantation

The Assessment of LEscol in Renal Transplantation (ALERT) study showed that long-term treatment (5–6 years) with fluvastatin (40—80 mg/day) non-significantly reduced the risk of coronary death or non-fatal MI, compared with placebo in ciclosporin treated kidney transplant recipients [14]. In the 2-year extension trial, fluvastatin led to a significant 35% relative reduction in the risk of cardiac death or non-fatal MI [15].

A Cochrane review looking at 22 studies in kidney transplant recipients found that statins may reduce major adverse cardiovascular events [16]. The adverse effect of statins, including on liver enzymes and creatine kinase, was uncertain [16].

Most statins are metabolised by the cytochrome P450 microsomal enzyme system. Concurrent therapy with inhibitors of this system, such as ciclosporin or tacrolimus, can lead to greater statin exposure and higher risk of side effects, such as rhabdomyolysis [17]. This risk appears to be greatest with simvastatin and lowest with fluvastatin or pravastatin.

Ezetimibe appears to be safe in kidney transplant recipients. It has been reported to interfere with ciclosporin levels; however, more recent reports suggest that this is unlikely to be a major clinical problem [18].

Fibrates have a high risk of side effects and are generally best avoided in kidney transplant recipients.

### Post-transplant diabetes mellitus and lipid lowering

Post-transplant diabetes mellitus (PTDM) affects 7–25% of people following kidney transplantation [19]. Conventional risk factors include age, obesity, and ethnicity. Transplant-related risk factors include corticosteroids, calcineurin inhibitors and acute rejection. There are no studies to guide lipid management in PTDM and, in the absence of specific evidence, it seems reasonable to use conventional lipid-lowering agents in combination with dietary and lifestyle advice to achieve lipid targets.

### Combined kidney pancreas transplant and lipid lowering

For people with type 1 diabetes and advanced DKD, simultaneous pancreas kidney transplantation (SPK) or pancreas after kidney transplantation (PAK) allows people to become insulin independent and has been shown to improve multiple markers of CVD [20]. All those being considered for SPK or PAK transplantation will have had prior indication for lipid-lowering therapy due to a cumulative lifetime risk of CVD. Therefore, unless there is an indication for discontinuation of lipid-lowering therapy, it would seem sensible to continue treatment of dyslipidaemia in this group.

We suggest that lipid-lowering agents be continued in those commencing dialysis, (Grade 2D).

We suggest that the decision to commence lipid-lowering agents de novo in those requiring dialysis (haemodialysis or peritoneal) should take into account risk of future atherosclerotic vascular events, life expectancy and, other comorbid disease (Grade 2D).

Where indicated, we recommend that lipid-lowering agents should be commenced post kidney transplantation or combined kidney-pancreas transplantation and that the choice and dose of lipid-lowering agent should take into account concurrent immunosuppressive therapy (Grade 1C).

Where indicated, we suggest that people who develop post-transplant diabetes mellitus are treated with lipid-lowering agents (Grade 2D).

### Recommendations for treatment targets

The 2010 CTT meta-analysis demonstrated that larger reductions in LDL cholesterol led to further reductions in major vascular events [3]. A lower LDL cholesterol (≤ 1.8 mmol/ L) was associated with a further 15% reduction in major vascular events [3]. There was no evidence of a threshold LDL cholesterol level or evidence of adverse effects with more intensive therapy.

We suggest the following treatment targets.

- TC ≤ 4.0 mmol/L.
- non-HDL cholesterol ≤ 2.5 mmol/L.
- LDL cholesterol ≤ 1.8 mmol/L (Grade 2D).

We have not suggested a percentage reduction for pragmatic reasons. Similarly, we have not suggested a graded approach to therapy with respect to risk stratification as we consider all those with diabetes (type 1 or 2) and DKD to be at high risk for CVD.

### Choice of lipid-lowering agent

#### Role for statins

Statins are 3-hydroxy-3-methylglutaryl-coenzyme A (HMG-CoA) reductase inhibitors and are the lipid modifying agent of choice for people with diabetes. Simvastatin is associated with a number of side effects and drug interactions with other agents (such as diltiazem and amlodipine). Thus, atorvastatin 20 mg is suggested as the first line lipid-lowering agent. Dose titration up to a maximum dose of 80 mg should be done with care, especially at lower eGFR < 30 mL/minute/1.73 m^2^.

Rosuvastatin is a high intensity statin. In CKD stage 1–2, there is no dose adjustment. In CKD stage 3 or below, the starting dose is 5 mg. The US Food and Drug Administration (FDA) advises that the maximum dose below eGFR < 30 mL/minute/1.73 m^2^ is 10 mg. However, European and SPC guidance is to avoid rosuvastatin at eGFR < 30 mL/minute/1.73 m^2^.

Where statin intolerance is an issue, consider switching to an alternate statin, reducing the dose or, alternate day dosage.

#### Role for ezetimibe

Ezetimibe blocks the intestinal absorption of cholesterol and upregulates hepatic LDL receptor expression, enabling reduction of atherogenic lipoproteins [21]. The main role for ezetimibe in DKD is as an adjunctive to statin use, or in statin intolerance. Ezetimibe can be used in mild to severe kidney disease and co-administered with any dose of statin.

#### Role for Bempedoic acid

Bempedoic acid is a once daily, oral medication used at a dose of 180 mg. It is a prodrug converted to bempedoyl-CoA by very-long-chain acyl-CoA synthetase-1, an enzyme present within the liver but absent in skeletal muscle, thus eliciting a liver specific action [22]. The active substrate, bempedoyl Co-A, inhibits ATP citrate lyase, an enzyme up stream of HMG-CoA reductase, thus suppressing cholesterol synthesis. This leads to increased membrane LDL receptors and LDL cholesterol clearance [22]. Elimination is mainly through renal, 70%, and hepatic clearance, 30%. Bempedoic acid has not been studied below eGFR 30 mL/min/1.73 m^2^.

NICE recommends Bempedoic acid for people with primary hypercholesterolaemia (heterozygous familial or non-familial) or mixed lipidaemia where statins are contraindicated or not tolerated and where ezetimibe alone is insufficient [26]. It is currently not recommended by NICE to be added where maximal statin therapy is insufficient [26].

#### Role for fibrates

Fibrates, peroxisome proliferator-activated receptor-α (PPAR-α) agonists, lower TG levels and TG rich particles. It has been proposed that TG rich particles participate in atherosclerosis. While CM and VLDL are too large to penetrate the arterial intima, the remnant particles are able to penetrate the intima and appear to reside for a longer period in the sub-intimal space. Thus, it would be reasonable to hypothesise that reducing TG levels would improve CVD risk.

Whilst there is no clear increase in progression to ESKD with fibrates, the reversible rise in creatinine which is reported consistently may in practice offset any perceived short-term advantage on albuminuria reduction. Addition of fibrates might be best restricted to younger people with fewer advanced complications and preserved GFR [27, 28]. Lower doses of fibrates, including fenofibrate and gemfibrozil, are recommended below eGFR 60 ml/min/1.73 m^2^. Fibrates should be withdrawn if eGFR falls below 30 ml/min/1.73 m^2^.

#### Role for PCSK9 inhibitors

Proprotein convertase subtilisin-kexin type 9 (PCSK9) is an endogenous hepatic LDL receptor ligand. Binding of PCSK9 to the LDL receptor leads to receptor degradation which prevents LDL receptor recycling. This leads to an increase in LDL.

PCSK9 monoclonal antibodies are administered by subcutaneous injection fortnightly or monthly. Inhibition of the binding of PCSK9 to the LDL receptor reduces LDL receptor degradation, and leads to significant reductions in LDL cholesterol.

Two PCSK9 inhibitors, alirocumab and evolocumab, have been approved by the European Medicines Agency (EMA) and FDA. Both drugs reduce LDL and non-HDL cholesterol in people with diabetes and may be useful for those unable to reach their cholesterol targets in combination with a statin or in people who are intolerant of statins.

The current recommendations for the use of PCSK9i set by NICE are set out in Table 2.

**Table 2:** Recommendations for use of PSCK9i (adapted from NICE) [42]

PCSK9i indications	Without CVD	With CVD	
		High risk^a^	Very high risk^b^
Primary non-familial hypercholesterolaemia or mixed dyslipidaemia		LDL ≥ 4.0 mmol/L	LDL ≥ 3.5 mmol/L
Primary heterozygous-familial hypercholesterolaemia	LDL ≥ 5.0 mmol/L	LDL ≥ 3.5 mmol/L	LDL ≥ 3.5 mmol/L

^a^ACS, CHD, PVD, ischaemic stroke, revascularisation

^b^Recurrent events in more than 1 vascular bed

#### Role for Inclisiran

Inclisiran is a small interfering RNA that prevents hepatic PCSK9 translation thus reducing LDL receptor degradation and increasing surface LDL receptors. It is injected subcutaneously at 0 months, 3 months and then every 6 months.

Inclisiran is primarily renally excreted and a third of the total administered dose is detectable in the urine after 24 h.

Inclisiran has been approved by the EMA and the FDA. NICE guidelines have placed it on the lipid lowering pathway for people with a history of ASCVD and raised LDL cholesterol, above 2.6 mmol/ L. It is currently not recommended for primary prevention. It has a lower threshold for approval compared to PCSK9i. In Wales, the guidelines differ. Inclisiran is licensed for people with high risk of CVD due to previous events and LDL cholesterol ≥ 4.0 mmol/L, those with recurrent disease and LDL cholesterol ≥ 3.5 mmol/L and, people with heterozygous familial hypercholesterolaemia and LDL cholesterol ≥ 5.0 mmol/L for primary prevention [33].

#### Role for Omega 3 fatty acids

Omega 3 fatty acids found in oily fish have been associated with reduced CVD risk in observational studies. However, formal randomised control trial evidence is mixed. Dietary fish oil supplements are poorly regulated and other low dose mixed preparations have failed to demonstrate cardiovascular benefit.

The Reduction of Cardiovascular Events with EPA—Intervention Trial (REDUCE-IT) was a phase 3b, double blind, placebo-controlled trial investigating 2 g icosapent ethyl twice daily in people on statins with established ASCVD or diabetes [43]. The primary endpoint was a composite of cardiovascular death, non-fatal myocardial infarction, non-fatal stroke, coronary revascularisation, or unstable angina. Primary end-point events occurred in 17.2% of the icosapent ethyl group, compared with 22.0% of the placebo group [43]. This was observed regardless of the presence of diabetes or level of eGFR. The possible mechanism of action may be related to reduced TG levels, reduced VLDL, plaque stability and anti-inflammatory effects.

Whilst icosapent ethyl is licenced for primary prevention for people with diabetes and at least one additional cardiovascular risk factor, NICE have only recommended its use for secondary prevention in people with established cardiovascular disease, taking a statin, and with TG > 1.7 mmol/L and LDL cholesterol between 1.04 and 2.60 mmol/L [45].

#### Role for Bile acid sequestrants

Bile acids sequestrants bind to bile acids in the gut and prevent their reabsorption. As bile acids are synthesized from cholesterol, this has a net effect of reducing cholesterol levels.

At the maximum dose, these reduce LDL cholesterol by up to 25%. However, they have adverse gastrointestinal effects and drug interactions limiting their use. They may also reduce the absorption of fat-soluble vitamins and other drugs. Colesevelam can be used in conjunction with statins.

### Recommendations

1. At all stages of DKD, we recommend initiation with statin therapy, atorvastatin 20 mg (Grade 1D).
2. In stage G1-G3a DKD, we recommend consideration of higher dose/intensity statin therapy for those who do not attain treatment targets on lower statin doses and recommend seeking specialist advice if eGFR < 30 ml/min/1.73 m^2^ (Grade 1D).
3. At all stages of DKD, we suggest consideration of submaximal statin and ezetimibe 10 mg combination therapy in those unable to tolerate higher statin doses (Grade 2B).
4. At all stages of DKD, in those with statin intolerance, we suggest ezetimibe 10 mg alone (Grade 2D).
5. In stage G1-G3a DKD, in those with statin intolerance, we suggest ezetimibe 10 mg in combination with Bempedoic acid 180 mg where treatment targets are not met (Grade 2D).
6. In stage G1–G3a DKD, we suggest that fenofibrate therapy (alone or in combination with statins) should only be used with specialist advice (Grade 2C).
7. In stage G3b–5 DKD, we recommend that there is no role for fibrates outside specialist care (Grade 1B).
8. We do not recommend fibrate ezetimibe combination therapy without specialist advice (Grade 1D).
9. We suggest consideration of inclisiran, in line with licensing and national guidelines, for secondary prevention in people who fail to achieve treatment targets. Currently there is limited data for use of inclisiran in severe DKD or ESKD; however, evidence exists for benefit up to stage G3b DKD (Grade 2C).
10. We suggest consideration of PCSK9 inhibitors, in line with licensing guidelines, in people who fail to achieve treatment targets. Currently there is limited data for use in severe DKD or ESKD; however, evidence exists for benefit up to stage G3b DKD (Grade 2C).
11. We suggest consideration of icosapent ethyl for secondary prevention, in line with licensing guidelines, in people with elevated fasted TG > 1.7 mmol/L and LDL cholesterol between 1.04 and 2.60 mmol/L. Currently there is limited data for use in severe DKD or ESKD (Grade 2C).

### Recommendations for the monitoring and safety of lipid-lowering agents

The overall safety of statins has been exhaustively evaluated. In general use, serious side effects are considered uncommon. Regarding the safety of statins in CKD, the 2009 Cochrane meta-analysis recorded no significant increase in the risk of rhabdomyolysis (defined as > 10 times the upper limit of normal (ULN)), nor in liver function abnormalities (defined as > 3 times the ULN) [46].

Women of childbearing potential should be advised about the teratogenic risks of statins. Women on statins and planning a pregnancy should stop this therapy three months before they attempt to conceive and should not restart until completion of breastfeeding [47].

The main side effects noted in RCTs for ezetimibe, fibrates, Bempedoic acid, inclisiran, PCKS9i and icosapent ethyl are listed in Table 3. For more extensive recommendations, please refer to the individual summary of product characteristics.

**Table 3:** Side effects of lipid-lowering agents

Drug	Side effect
Ezetimibe	• Tiredness • Joint pain • Upper respiratory tract infection • Diarrhoea
Fibrate	• Abdominal pain • Diarrhoea, constipation • Nausea and vomiting
Bempedoic acid	• Increased risk of gout • Slight reduction in eGFR • Should not be used in severe liver disease
Inclisiran	• Site specific reactions • Non-specific symptoms such as headache and fatigue
PCSK9i	• Nasopharyngitis • Myalgia • Upper respiratory tract infection
Icosapent ethyl	• Allergy warnings listed to fish, shellfish, soya, and peanuts • Atrial fibrillation • Serious bleeding events • Peripheral oedema

### Recommendation for when to stop lipid-lowering agents

CVD is prevalent in older people; however, evidence for risk reduction by lipid management is limited. Subgroup analysis of major statin trials have been performed to determine if there is a differential outcome among different age groups. A CTT analysis of statin therapy at different ages found evidence of benefit in those aged > 75 years [48]. There may, however, be an inherent bias in this meta-analysis and indeed other studies and post-hoc analyses as people with frailty, for example those with dementia or multiple comorbidities, would be unlikely to be recruited to these trials. The older group of participants included may therefore represent the healthier and possibly more engaged cohorts. A case-by-case approach is recommended for cessation of therapy in the elderly.

## Data Availability

No datasets were generated or analysed during the current study.
